# A targeted CRISPR screen identifies ETS1 as a regulator of HIV-1 latency

**DOI:** 10.1371/journal.ppat.1012467

**Published:** 2025-04-08

**Authors:** Manickam Ashokkumar, Terry L. Hafer, Abby Felton, Nancie M. Archin, David M. Margolis, Michael Emerman, Edward P. Browne

**Affiliations:** 1 Department of Medicine, University of North Carolina at Chapel Hill, Chapel Hill, North Carolina, United States of America; 2 UNC HIV Cure Center, University of North Carolina at Chapel Hill, Chapel Hill, North Carolina, United States of America; 3 Division of Basic Sciences, Fred Hutchinson Cancer Center, Seattle, Washington, United States of America; 4 Division of Human Biology, Fred Hutchinson Cancer Center, Seattle, Washington, United States of America; 5 Department of Microbiology and Immunology, University of North Carolina at Chapel Hill, Chapel Hill, North Carolina, United States of America; Loyola University Chicago, UNITED STATES OF AMERICA

## Abstract

Human Immunodeficiency virus (HIV) infection is regulated by a wide array of host cell factors that combine to influence viral transcription and latency. To understand the complex relationship between the host cell and HIV-1 latency, we performed a lentiviral CRISPR screen that targeted a set of host cell genes whose expression or activity correlates with HIV-1 expression. We further investigated one of the identified factors - the transcription factor ETS1, and found that it is required for maintenance of HIV-1 latency in both latently infected cell lines and in a primary CD4 T cell latency model. Interestingly, ETS1 played divergent roles in actively infected and latently infected CD4 T cells, with knockout of ETS1 leading to reduced HIV-1 expression in actively infected cells, but increased HIV-1 expression in latently infected cells, indicating that ETS1 can play both a positive and negative role in HIV-1 expression. CRISPR/Cas9 knockout of ETS1 in CD4 T cells from ART-suppressed people with HIV-1 (PWH) confirmed that ETS1 maintains transcriptional repression of the clinical HIV-1 reservoir. Transcriptomic profiling of ETS1-depleted cells from PWH identified a set of host cell pathways involved in viral transcription that are controlled by ETS1 in resting CD4 T cells. In particular, we observed that ETS1 knockout increased expression of the long non-coding RNA MALAT1 that has been previously identified as a positive regulator of HIV-1 expression. Furthermore, the impact of ETS1 depletion on HIV-1 expression in latently infected cells was partially dependent on MALAT1. Additionally, we demonstrate that ETS1 knockout resulted in enhanced abundance of activating modifications (H3K9Ac, H3K27Ac, H3K4me3) on histones located at the HIV-1 long terminal repeat (LTR), indicating that ETS1 regulates the activity of chromatin-targeting complexes at the HIV-1 LTR. Overall, these data demonstrate that ETS1 is an important regulator of HIV-1 latency that impacts HIV-1 expression through repressing MALAT1 expression and by regulating modification of proviral histones.

## Author summary

HIV-1 latency is a major obstacle for the eradication of HIV-1. However, the molecular mechanisms that restrict proviral expression during therapy are not well understood. Identification of host cell factors that silence HIV-1 would create opportunities for targeting these factors to reverse latency and eliminate infected cells. Our study aimed to explore mechanisms of latency in infected cells by employing a lentiviral CRISPR screen and CRISPR/Cas9 knockout in primary CD4 T cells. These experiments revealed that ETS1 is essential for maintaining HIV-1 latency in primary CD4 T cells and we further confirmed ETS1’s role in maintaining HIV-1 latency through CRISPR/Cas9 knockout in CD4 T cells from antiretroviral therapy (ART)-suppressed individuals with HIV-1. Furthermore, ETS1 depletion increased the abundance of activating histone modifications at the HIV-1 LTR promoter. Overall, our study demonstrates that ETS1 is a critical regulator of HIV-1 latency.

## Introduction

Despite the development of successful antiretroviral therapy (ART) that can reduce circulating human immunodeficiency virus (HIV-1) plasma viral loads to undetectable levels, the virus rebounds rapidly from a highly stable latent proviral reservoir following ART interruption [1]. Latently infected cells with HIV-1 are thus a major obstacle for the eradication of virus from people with HIV-1 (PWH). Toward this goal, several different classes of latency reversing agents (LRAs) have been developed and investigated for their ability to reactivate latently infected cells and promote their killing by the immune system or by viral cytopathic effect [2–4]. However, none of the current LRAs that have been tested *in vivo* have proven to be effective in reducing the size of the HIV-1 reservoir. The reasons for this lack of an impact on the clinical reservoir are unclear, but it is likely the result of the complex relationship between the virus and the set of cellular transcription factors (TFs) that regulate HIV-1. Individual LRAs that target single pathways are typically inefficient, reflecting the heterogeneous nature of the reservoir, and suggesting that combinations of LRAs will likely be required to achieve broad viral reactivation.

The HIV-1 long terminal repeat (LTR) contains DNA binding sites for several ubiquitously expressed and regulated cellular TFs with activating or repressive functions [5]. In recent years, transcriptional regulators (activators or repressors) have been identified which interact directly or indirectly with the HIV-1 5’LTR, and the combined activity of these factors determines the fate of HIV-1 transcription and latency. Nevertheless, the regulation of the viral promoter is still incompletely understood and additional regulatory factors likely exist. Identification of these factors and the characterization of their roles in the maintenance of the HIV-1 reservoir will likely lead to the development of new tools for clinical latency reversal.

We have recently used combined single cell multi-omic profiling of a primary CD4 T cell model of HIV-1 latency to identify a set of host cell genes whose expression or activity correlates with HIV-1 RNA levels [6]. Based on this observation we hypothesized that some of these HIV-correlated factors could represent novel regulators of HIV-1 expression. Genome editing platforms based on the clustered regularly interspaced palindromic repeat (CRISPR)/CRISPR associated protein 9 (Cas9) system have been recognized as promising tools for probing interactions between the HIV-1 proviral genome and the host cell. Several studies using CRISPR/Cas9 technology have previously identified cellular factors that can affect HIV-1 replication or latency [7–10]. In this study we have used a set of HIV-1 correlated genes to conduct a targeted CRISPR screen for novel latency regulating factors. Specifically, we have carried out a lentiviral CRISPR-Cas9 pooled knockout screen in J-Lat 10.6 cells, a Jurkat cell-based model of HIV-1 latency. This approach revealed several novel genes involved in latency, including the transcriptional regulator, ETS1 (E26 transformation–specific-1). In further validation experiments in primary CD4 T cells and in cells from people with HIV-1, we found that ETS1 has a complex role in establishing latency and reactivation, playing a positive role in actively infected CD4 T cells, but a repressive role in latently infected CD4 T cells. Furthermore, we determined that ETS1 suppresses HIV-1 expression by inhibiting expression of the long non-coding RNA MALAT1 and by preventing the addition of transcription-enhancing modifications to provirus-associated histones.

## Results

### Targeted CRISPR screen design

**Fig 1:**
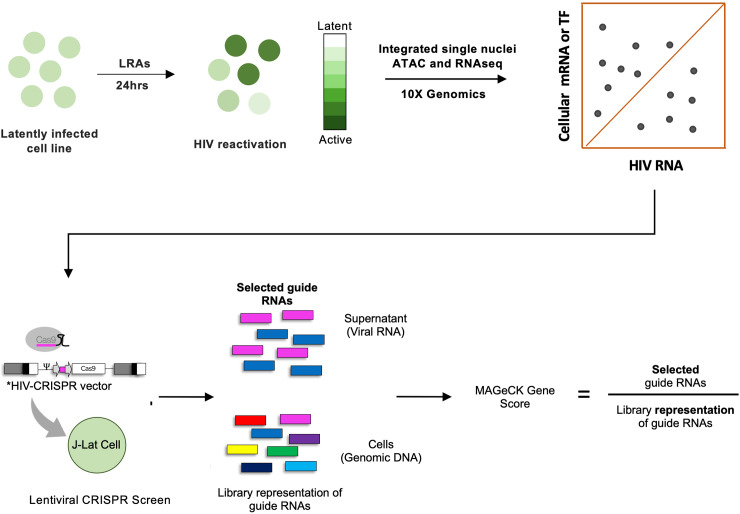
Schematic of targeted CRISPR screen for novel latency regulating factors. In this study we conduct a targeted CRISPR screen for novel latency regulating factors. HIV-1 correlated factors were first identified using a prior dataset and a 351 target pooled gRNA library (TxLatent) was constructed using the HIV-CRISPR vector that contains an HIV-1 RNA packaging signal. Screening was carried out in J-Lat 10.6, a Jurkat cell-based model of HIV-1 latency. gRNAs that reactivate or inhibit HIV-1 expression are enriched or depleted in secreted virions found in the cellular supernatant respectively. Screening was also carried out in the presence or absence of four different latency reversing agents (LRAs).

We have previously shown that HIV-1 expression in CD4 T cells is correlated with expression of a set of host cell genes [6]. We hypothesized that a subset of these genes could also be important for controlling HIV-1 expression and latency. Thus, we sought to examine the role of these HIV-correlated genes (S1 Table) using a previously established HIV-CRISPR screening system [11–13]. A schematic description of the screening approach is shown in **Fig 1A**. This screening approach uses pooled lentiviral sgRNAs against a set of target genes in a Jurkat-derived cell line with latent proviruses that encodes the HIV-1 Gag protein (J-Lat 10.6 cells) [14,15]. A key aspect of the HIV-CRISPR screen is that the lentiviral guide RNAs (gRNAs) contain an HIV-1 psi (ψ) packaging signal and are thus packaged *in trans* into virions in cells with reactivated HIV-1 gene expression. Thus, if a gene knockout affects viral gene expression, this sgRNA will be either depleted or enriched in the supernatant of the J-Lat 10.6 cells. We first generated an sgRNA library targeting these 351 genes of interest, as well as a set of positive and negative controls, hereafter referred to as the TxLatent library. The target set for this screen was highly enriched for cellular transcriptional regulators. These guide RNAs (8 per gene along with 150 non-targeting guides) were cloned into an HIV-CRISPR vector, transfected as a pool into HEK293T cells to generate stocks of the HIV-CRISPR particles, then used to transduce the J-Lat 10.6 cell line. After puromycin selection for transduction by the HIV-CRISPR vector, these cells were stimulated in parallel with limiting concentrations of four different latency reversing agents (LRAs) or with an equivalent volume of vehicle (dimethyl sulfoxide, DMSO) to look for synergistic or dependent relationships between these compounds and individual knockouts. The LRAs used were AZD5582 (non-canonical NF-κB agonist), prostratin (protein kinase C agonist), vorinostat (histone deacetylase inhibitor) and iBET151 (bromo-and extra-terminal domain inhibitor) [6]. After 24h of LRA stimulation, we harvested genomic DNA and viral supernatants to assess how each gene knockout affected viral production by next-generation sequencing. We also validated reactivation of J-Lat 10.6 cells upon LRA treatment by performing a reverse transcriptase (RT) assay on supernatant from the library transduced cells (S1 Fig).

**Fig 2:**
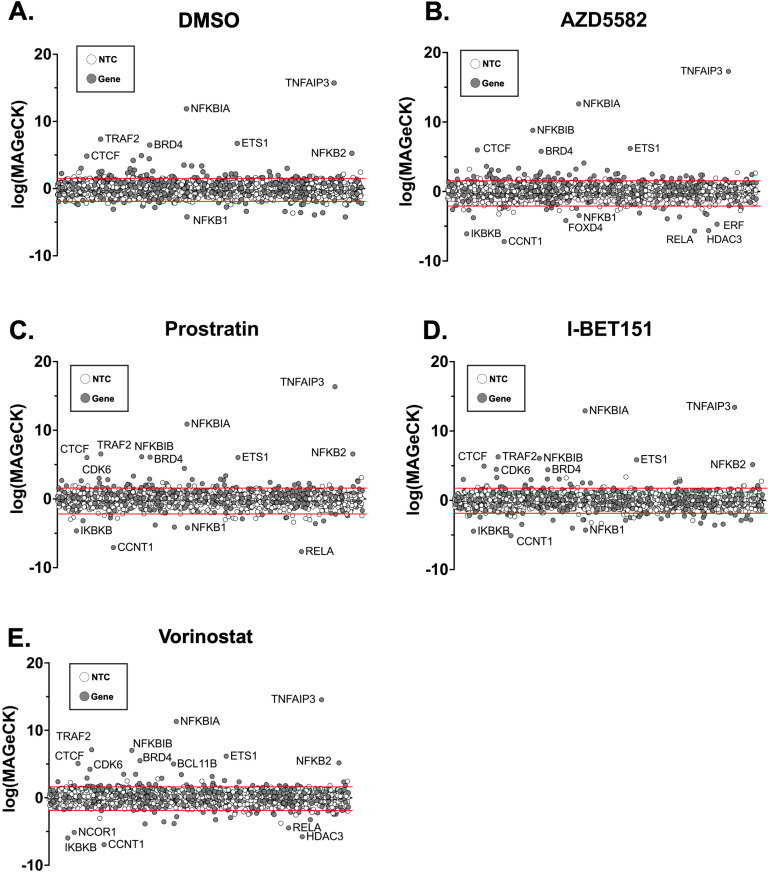
CRISPR Screen of TxLatent library. Latency HIV-CRISPR screen data is displayed for J-Lat 10.6 cells transduced with TxLatent library and subsequently treated with DMSO **(A)**, AZD5582 (10nM) **(B)**, prostratin (75nM) **(C)**, iBET151 (75nM) **(D)**, or vorinostat (500nM) **(E)**. Genes in the TxLatent library are randomly displayed on the x-axis, in the same order across each treatment. The -Log(MAGeCK Score), a statistical pipeline that takes into account the enrichment/depletion of all guides for a given gene as well as the variation between replicates [16] is plotted for the enriched genes and the Log(MAGeCK score) is plotted for depleted genes, and combined together to display on a single graph for each treatment. A list of “synthetic” NTCs (synNTCs) was created by randomly grouping sets of 8 NTCs and used to generate a cutoff for gene hits that were enriched or depleted. SynNTCs are shown with white circles and genes targeted by gRNAs in the TxLatent library are shown with gray circles. The red lines on each graph represent the two standard deviations of mean of the synNTCs: DMSO, 1.52 and -1.91; AZD5582, 1.53 and -2.10; iBET151, 1.75 and -1.87; prostratin, 1.58 and -2.19; and vorinostat, 1.61 and -1.9.

We assessed the degree that guides (aggregated by gene) are either enriched or depleted relative to the non-targeting controls (NTCs) by determining the Model-based Analysis of Genome-wide CRISPR-Cas9 Knockout (MAGeCK) scores for each gene [16], which takes into account the degree of enrichment or depletion of all 8 guides in the RNA of the supernatant versus the DNA in the cell [12] (Fig 2A-2E, and S2 Table). First, we examined the depleted genes whose knockout prevented viral gene expression during latency reversal in J-Lat 10.6 cells. Consistent with a previous targeted CRISPR screen using this system, *CCNT1*, which encodes Cyclin T1, (Fig 2B-2E) was among the most depleted targets in J-Lat cells treated with an LRA [13], and *CCNT1* knockout inhibited latency reversal under all conditions tested. Similarly, *IKBKB* (the inhibitor of NF-κB Kinase) is a positive regulator of the NF-κB signaling pathway and is also consistently depleted in each LRA treatment (Fig 2C-2E). Curiously, guides targeting *HDAC3* were also depleted in some conditions (AZD5582; **Fig 2B** and vorinostat; **Fig 2E**), but not in others. Overall, these data confirm that the library and screen design could reliably identify HIV-1 regulating factors in these cells.

We then focused on genes that were positively enriched in this screen (e.g. genes whose knockout caused HIV-1 to be released from latency) since these could represent targets for potential latency reversing agents in a “shock and kill” strategy. We observed a substantial number of gene hits enriched in the DMSO (56 genes), AZD5582 (69 genes), iBET151 (28 genes), prostratin (55 genes), and vorinostat (48 genes) conditions. Among the most highly enriched hits in cells treated with the DMSO control (**Fig 2A**), as well as with each LRA treatment, were several negative regulators of the NF-κB signaling. Notably, the top enriched gene for each treatment condition was *TNFAIP3* (Fig 2A-2E). *TNFAIP3* encodes the Tumor Necrosis Alpha Induced Protein 3, also known as A20, and has been shown to be a key negative regulator of NF-κB signaling by several groups [17,18]. Moreover, *NFKBIA* and *NFKBIB* (NF-κB inhibitors Alpha and Beta, respectively) targeting sgRNAs are consistently positively enriched in secreted virions with each LRA treatment (Fig 2B-2E). These genes encode proteins that negatively regulate NF-κB signaling by sequestering NF-κB in the cytoplasm [19]. Interestingly, a gene encoding a members of the cohesin complex (CTCF) was identified in our analysis as playing a role in repression of HIV-1 (**Fig 2**, and S2 Table), consistent with our previous observations [20]. CTCF plays a role in forming a complex that is responsible for mediating long range DNA interactions [21,22], potentially suggesting a role for this complex or three-dimensional chromatin structure in HIV-1 transcriptional regulation.

We also observed several novel hits, including the transcriptional regulator *ETS1* and the cyclin-dependent kinase *CDK6*. *CDK6* targeting gRNAs were also enriched in the supernatant for prostratin, iBET151 and vorinostat treatments, indicating a role in HIV-1 expression (Fig 2C-2E). CDK6 has not previously been implicated as a latency regulating factor, though other CDKs such as CDK9 and CDK7 have well-described roles in the regulation of RNApol2 [23]. *ETS1* targeting gRNAs were enriched in DMSO exposed cells and for all LRA conditions indicating a general role in repression of HIV-1. Curiously, previous work has identified ETS1 as a potential regulator of HIV-1, but these reports suggested that ETS1 cooperates with NF-κB to enhance transcriptional activity of HIV-1 enhancers at the LTR [24,25]. Nevertheless, in this screen, we observed an apparent negative regulatory role for ETS1 in HIV-1 expression.

### Primary CD4 T cell CRISPR validation for the role of novel factors in HIV-1 latency

**Fig 3:**
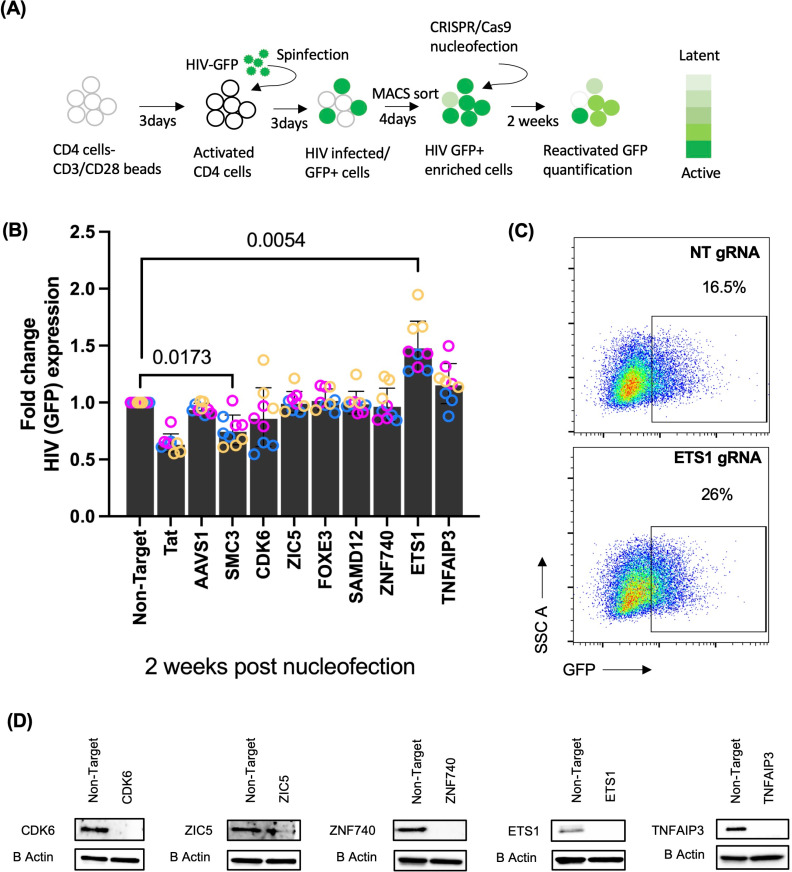
CRISPR validation of targets in HIV-1 infected primary CD4 T cells. **(A)** Schematic overview of experimental design for CRISPR-Cas9 targeting of host genes in HIV-GFP infected primary CD4 T cells. Activated CD4 T cells were infected with HIV-GFP [26]. At 3 days post infection, infected (GFP+) cells were enriched by magnetic sorting and cultured for 4 days before nucleofection of Cas9/gRNA complexes targeting AAVS1, SMC3, CDK6, ZIC5, FOXE3, SAMD12, ZNF740, ETS1, and TNFAIP3 or a non-targeting gRNA (NT). Pools of 3 different gRNAs were used for each target. HIV-GFP infected cells are denoted in green color. Differential intensity of green color represents latent and active infection. **(B)** Relative level of HIV-1 expression measured as GFP using flow cytometry. GFP expression was measured at two weeks post nucleofection. Data are displayed as GFP expression in fold change normalized to cells nucleofected with NT gRNA. Each condition represents three biological replicates/donors (Yellow, Blue and Purple) and three technical replicates for each donor. **(C)** Dot plots representing viral GFP expression at two weeks post knockout. **(D)** Western blot of the host cellular factors targeting CDK6, ZIC5, ZNF740, ETS1, and TNFAIP3 or a control non-targeting (NT) Cas9/gRNA nucleofected primary cells at one week post nucleofection (pKO). Statistical analysis was conducted for cumulative data. Error bars represent standard deviations, and P values displayed were determined by two-way ANOVA Tukey’s multiple comparisons test. WT, wildtype; NT, non-target; CRISPR, clustered regularly interspaced short palindromic repeats; Cas9, CRISPR-associated protein 9.

From the candidate HIV-repressing genes identified by the lentiviral CRISPR screen in the JLat 10.6 cell line model, we selected 10 significantly enriched host cellular genes/transcription factors, including AAVS1, CDK6, ETS1 FOXE3, SAMD12, SMC3, TNFAIP3, ZIC5 and ZNF740, for further validation in a primary CD4 T cell latency model (**Fig 3A**). In this model, primary CD4 T cells from seronegative donors are activated via their TCR, then infected with a GFP-expressing reporter strain of HIV-1 (HIV-GFP) [26,27] followed by enrichment of infected cells at 3 days post infection. After infection, the cells were maintained in culture with IL-2 for up to three weeks, during which time viral gene expression progressively declines as the cells revert to a resting state, and a population of latently infected (GFP-) cells emerges [20,27–30]. Three days post enrichment, cells were nucleofected with Cas9/sgRNA ribonucleoprotein (RNP) complexes designed against each target. RNPs with scrambled non-targeting sgRNAs or Tat-targeting sgRNAs were nucleofected in parallel as negative and positive controls respectively (**Fig 3A**). Viral gene expression was then measured by flow cytometry for GFP at 2 weeks post nucleofection to determine the effect on HIV-1 latency (**Fig 3B**). As expected, CRISPR targeting of Tat led to a potent reduction in viral expression. Interestingly, viral expression was significantly elevated for cells in which ETS1 was targeted (p < 0.0001) compared to NT control (**Fig 3B** and **3C**). The efficiency of target knockout for most cellular targets was quantified using western blot analysis (**Fig 3D**). However, for three targets (SAMD12, SMC3, and FOXE3) we were unable to confirm either expression or depletion of the target. Nevertheless, these data indicate that, as the infected CD4 T cells return to a resting state, ETS1 plays an important role in establishing repression of latent HIV.

### ETS1 promotes HIV-1 expression in actively infected cells and inhibits HIV-1 expression in latently infected cells

**Fig 4:**
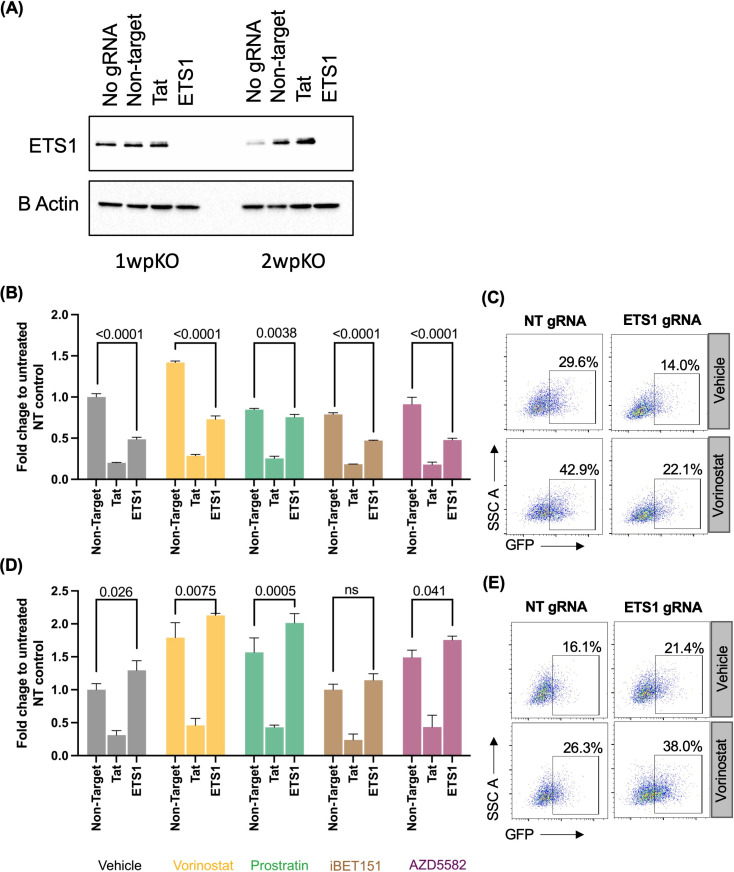
ETS1 knockout in primary CD4 T cells reveals positive and negative roles in HIV-1 expression. Primary CD4 T cells were activated, infected with HIV-GFP, enriched and nucleofected as in Fig 3. **(A)** Western blot of the ETS1-targeting or a control non-targeting (NT) Cas9/gRNA nucleofected primary cells for beta actin and ETS1 expression at one and two weeks post nucleofection (pKO). **(B-E)** Flow cytometry of infected cells without stimulation or after 24 h of stimulation with one of four different LRAs (vorinostat, prostratin, iBET151, or AZD5582) at one **(B)** and two **(D)** week post nucleofection. **(C** and **E)** Dot plots representing viral GFP expression at one and two weeks post knockout respectively. Data are represented as fold change in percent GFP expression, normalized to cells nucleofected with NT gRNA. Error bars represent standard deviation, and P values displayed were determined by two-way ANOVA Tukey’s multiple comparisons test. Data shown are representative of two independent experiments with different donors, with biological triplicates for each condition.

ETS1 has previously been reported to positively regulate HIV-1 expression in complexes with other cellular transcription factors, like NF-κB, NFAT, and USF-1 [25,31]. An ETS1 binding site has been identified in the LTR distal region, and other ETS family members have also been shown to bind the LTR [32]. To reconcile these previous observations with our finding that ETS1 participates in repressing latent HIV-1, we examined the impact of ETS1 knockout at different timepoints post infection as well as in the presence of latency reversing agents (LRAs) (**Fig 4**). In our latency model, T cells are first activated through TCR-mediated signaling, and slowly return to a resting state over two weeks, during which time HIV-1 expression declines as the cells undergo transcriptional and metabolic reprogramming [27–30]. RNPs with sgRNAs targeting ETS1, the HIV-1 Tat transcriptional regulator or a non-targeting control were electroporated into recently activated infected primary cells to examine the impact on HIV-1. Western blot analysis of nucleofected cells confirmed nearly complete depletion of ETS1 at 1- and 2-week post nucleofection (**Fig 4A**). Strikingly, when we examined an early timepoint (1wpi) post infection, we observed that ETS1 depleted cells had reduced HIV-1 expression compared to non-targeting controls (**Fig 4B** and **4C**), while at later timepoints (2wpi) we observed an increase in HIV-1 expression for ETS1 depleted cells, relative to controls (**Fig 4D** and **4E**), consistent with our previous observation (**Fig 3B**). Thus, ETS1 appears to play distinct roles at different times post T cell activation – a positive role at earlier times while the cells are still in a recently activated state, but a repressive role at later timepoints when the cells have returned to a resting state.

We also evaluated the role of ETS1 in latently infected primary CD4 T cells in response to different LRAs that we used for the initial screen in J-Lat 10.6 cells. Specifically, we stimulated the infected cells with a panel of LRAs with distinct mechanisms of action: vorinostat (HDACi), prostratin (PKCa), iBET151 (BETi), and AZD5582 (non-canonical NF-κB agonist) at 500nM for 24h. At 1wpi this stimulation caused a significant increase in the percentage of GFP+ cells in the cell population for vorinostat but caused only a small response to prostratin and no apparent response to iBET151 or AZD5582. However, at two weeks post nucleofection, depletion of ETS1 caused augmented viral gene expression as expected in all drug conditions. At 2wpi, both ETS1 depleted cells and control cells also responded to vorinostat, prostratin, and to AZD5582 but not iBET151. These data indicate that ETS1 is important cellular host factor for both expression of HIV-1 in active cells and establishment of latent HIV-1 infection in resting cells, but that ETS1 depletion does not noticeably change the sensitivity of latent proviruses to benchmark LRAs.

### ETS1 expression is broadly required for repression of HIV-1 in latently infected cell lines and in latently infected primary CD4 T cells

**Fig 5:**
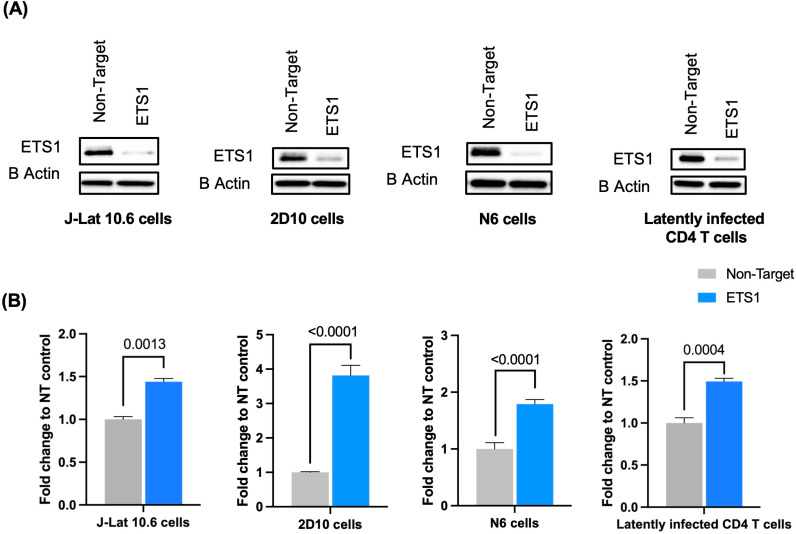
ETS1 knockout reactivates HIV-1 transcription in cell line models of HIV-1 latency and in latently infected primary CD4 T cells. CRISPR knockout was carried out in three latently infected cell lines, J-Lat 10.6, 2D10, and Jurkat-N6 cells, and in latently infected CD4 T cells *in vitro* with non-targeting or ETS1 targeting gRNAs. Cells harvested at 1 week post nucleofection were quantified for ETS1 depletion and HIV-1 reactivation using western blot and flow cytometry, respectively. **(A)** Depletion of ETS1 in nucleofected J-Lat 10.6, 2D10, Jurkat-N6 cells, and latently infected primary CD4 T cells was analyzed by western blot. **(B)** Relative GFP expression in latency cell line models, and in latently infected CD4 T cells nucleofected with ETS1-targeting or NT control Cas9/gRNA complexes. GFP expression was normalized to cells nucleofected with non-target gRNA and presented as fold change. Experiment was conducted with three biological replicates for each condition. Error bars represent standard deviation, and P values displayed were determined by two-way ANOVA Tukey’s multiple comparisons test.

To further examine the role of ETS1 as a repressor of HIV-1 expression in latently infected cells, we next examined whether ETS1 is required for maintaining HIV-1 latency in three different cell line models of latency. To achieve this, we performed CRISPR-mediated knockout of ETS1 in three different latently infected cell lines, J-Lat 10.6 cells [14,15], 2D10 cells [33], and Jurkat-N6 cells [34] with non-targeting or ETS1 targeting gRNAs. Both 2D10 cells and J-Lat 10.6 cells contain a quiescent integrated HIV-1 provirus in which a viral gene has been replaced by coding sequence for enhanced GFP (eGFP). Jurkat-N6 cells contain a full-length replication competent provirus derived from the NL4-3 strain of HIV-1 in which the Nef gene has been replaced by coding sequence for the murine CD24 transmembrane protein. Cells harvested at 1 week post nucleofection were quantified for ETS1 depletion and HIV-1 reactivation by western blot and flow cytometry for GFP (for 2D10 cells and J-Lat 10.6 cells) or for murine CD24 (for Jurkat-N6 cells), respectively. We observed efficient depletion of ETS1 in cells electroporated with ETS1-targeting guide RNAs for all cell lines (**Fig 5A**). As expected, we observed significant HIV-1 reactivation in the ETS1 depleted latently infected cell lines, with elevated relative HIV-1 reactivation levels ranging at 1.4, 3.8, and 1.8-fold change for J-Lat 10.6, 2D10, and Jurkat-N6 cells, respectively (**Fig 5B**) compared to non-targeting (NT) guide RNAs with statistical significance (p = 0.0013, <0.0001, and <0.0001, respectively; 2-way ANOVA Tukey’s multiple comparisons test). In addition, we performed CRISPR-mediated knockout of ETS1 in *in vitro* generated latently infected primary CD4 T cells. Latently infected primary CD4 T cells were generated as described in the methods section. We found that depletion of ETS1 in latently infected primary CD4 T cells significantly (p = 0.0004) reactivates HIV-1 from latency (**Right panel, Fig 5A and 5B**). These findings confirm that ETS1 is required for the maintenance of latent HIV-1 infection in several cell line models of HIV-1 latency and in latently infected primary CD4 T cells.

### ETS1 is required for repression of latent HIV-1 in cells from ART-suppressed people with HIV-1

**Fig 6:**
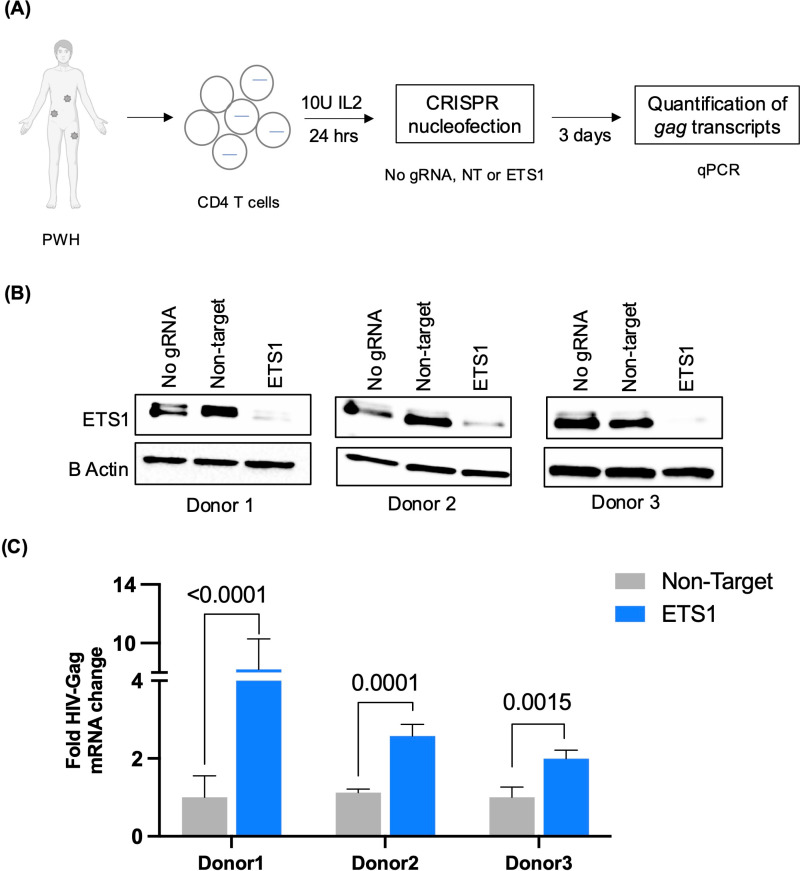
ETS1 knockout reactivates HIV-1 transcription in CD4 T cells from people with HIV-1 (PWH) on antiretroviral therapy (ART) *ex vivo.* **(A)** Schematic of *ex vivo* CRISPR nucleofection experiment in resting CD4 T cells isolated from PWH on ART. CRISPR nucleofection of ETS1-targeting or non-targeting (NT) Cas9/gRNAs was performed after a 24 h culture of PWH CD4 T cells followed by quantification of Gag RNA expression at 3 days post nucleofection by quantitative PCR. **(B)** Depletion of ETS1 in nucleofected resting CD4 T cells was analyzed by western blot. **(C)** Relative abundance of Gag viral RNA expression in CD4 T cells nucleofected with ETS1-targeting or NT control Cas9/gRNA complexes. Gag viral RNA expression was normalized to cells nucleofected with non-targeting gRNA and presented as fold change. Experiment was conducted in three biological replicates/donors with three technical replicates for each condition. Error bars represent standard deviation, and P values displayed were determined by two-way ANOVA Tukey’s multiple comparisons test. Figure elements prepared using Biorender.

Having found that ETS1 contributes to suppressing HIV-1 expression in different cell line models of HIV-1 latency, and in resting CD4 T cells that had been infected *ex vivo*, we next examined whether ETS1 is required for maintaining HIV-1 latency in the clinical reservoir. To investigate this, CD4 T cells were isolated from three ART-suppressed people with HIV-1 (PWH). Isolated total CD4 T cells were rested for 24 h followed by nucleofection with non-targeting or ETS1 targeting gRNAs. A schematic of CRISPR/Cas9 based nucleofection in CD4 T cells from PWH is depicted in **Fig 6A**. At 3 days post nucleofection, complete depletion of ETS1 was seen in cells electroporated with ETS1-targeting guide RNAs by western blot (**Fig 6B**). To determine the effect of ETS1 depletion on HIV-1 expression, we then carried out quantitative PCR for Gag unspliced viral RNA for each sample. Notably, we observed significant upregulation of HIV-1 Gag mRNA expression within the ETS1 depleted cells for all three donors, with elevated expression levels ranging at 8.2, 2.3, and 2-fold change (**Fig 6C**) compared to non-targeting (NT) guide RNAs with statistical significance (p < 0.0001, 0.0001, and 0.0015, respectively; 2-way ANOVA Tukey’s multiple comparisons test). These findings further establish that ETS1 is required for the maintenance of latency for the clinical HIV-1 reservoir.

### Transcriptomic profiling of ETS1 depleted CD4 T cells identifies several known and novel candidate regulators of HIV-1 gene expression

**Fig 7:**
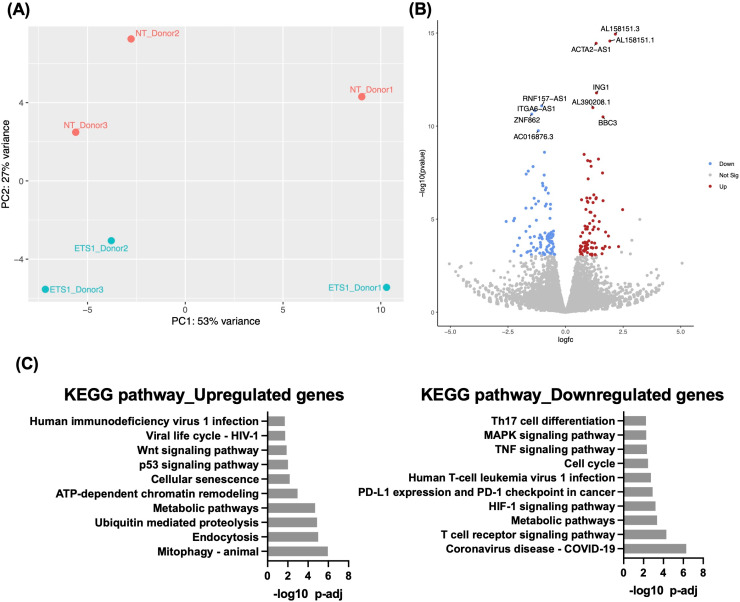
Analysis of differential gene expression in CD4 T cells from PWH on ART after depletion of ETS1. CD4 T cells from three PWH on ART were nucleofected with Cas9/gRNA complexes targeting ETS1 or non-targeting control (NT) as described in Fig 6, followed by bulk RNAseq. **(A)** Principal component analysis (PCA) plot of samples based on gene expression data for each sample. ETS1-targeted samples shown by blue dots, non-targeting gRNA samples by red dots. Each data point represents an individual sample. The y-axis and x-axis represent the first and second principal components, respectively. **(B)** Volcano plot of overall differentially expressed genes (DEGs) between HIV-1 infected CD4 T cells nucleofected with ETS1 targeting sgRNA/Ca9 complexes vs non-targeting. **(C)** Database for Annotation, Visualization and Integrated Discovery (DAVID) analysis of upregulated and downregulated genes of the RNA-sequencing analysis of ETS1 vs control cells. The 10 most significant KEGG pathways are shown.

Although our data demonstrate that ETS1 can play a suppressive role in HIV-1 expression and helps to maintain silencing of the HIV-1 reservoir, the mechanisms by which ETS1 contributes to HIV-1 latency and reactivation in PWH are not characterized. To further understand the role of ETS1 in regulation of HIV-1 expression, we analyzed the transcriptome of CD4 T cells from three virally suppressed PWH on ART with and without *ex-vivo* knockout of ETS1. RNA from the ETS1 targeting or non-targeting sgRNA nucleofected PWH donors (**Fig 6A**) was analyzed by bulk RNA sequencing (RNAseq). Principal component analysis (PCA) was used to determine the overall impact of ETS1 knockout on gene expression patterns across the conditions. Importantly, the samples clustered by knockout condition, indicating a consistent transcriptomic impact of ETS1 knockout across the donors (**Fig 7A**). We identified several differentially expressed genes (DEGs) between the two conditions, 178 genes total (p_adj_ < 0.05), with 86 genes upregulated and 92 downregulated (**Fig 7B**, and S3 Table).

Interestingly ETS1 was not identified as a DEG by this analysis, despite being clearly depleted by western blot, prompting us to examine ETS1 mapping reads in the dataset in more detail. For ETS1 targeting, three ETS1-specific sgRNAs were used in combination (sgRNA1, sgRNA2, and sgRNA3). When we examined read coverage between the recognition sites for sgRNA1 and sgRNA2 we observed a clear drop in read coverage (S2A Fig), indicating that the sgRNAs caused a disruption to the ETS1 gene within this region, but likely do not result in nonsense-mediated decay [35–37]. Thus, although the overall number of RNAseq reads within ETS1 did not change with sgRNA targeting, the gene is clearly affected by the sgRNAs, leading to reduced ETS1 protein expression.

To understand the overall impact of ETS1 knockout on CD4 T cells from PWH, we next examined the pathways represented within the set of DEGs. Interestingly, some of the top upregulated transcripts are involved in mitophagy, endocytosis, metabolic pathways, and ATP-dependent chromatin modeling (**Fig 7C, Left panel**). ATP-dependent chromatin-remodeling complexes play an important role in HIV-1 transcription as these complexes are recruited by HIV-1 Tat to the long terminal repeat (LTR) to facilitate active transcription [38]. Notably, ETS1 depletion also upregulated genes involved in the Wnt signaling pathway, HIV-1 viral life cycle, and HIV-1 infection pathways (**Fig 7C**). Downregulated pathways included ‘COVID-19’, ‘T cell receptor signaling pathway’, ‘metabolic pathway’, and ‘HIF-1 signaling pathway’ (**Fig 7C Right panel**). HIF-1 signaling is involved in viral replication and inflammation through extracellular vesicles [39]. ETS1 depletion also downregulated transcripts involved in MAPK signaling, TNF signaling, and Th17 cell differentiation that are important in several cellular responses, including expression of several inflammatory cytokines.

Expression of the ING1 (Inhibitor Of Growth Family Member 1) was upregulated in response to ETS1 depletion. ING1 is a nuclear protein involved in Chromatin Regulation that physically interacts with p53/TP53 in the negative regulatory pathway of cell growth by modulating p53-dependent transcriptional activation. Transcriptional activators KLF7 and FOXH were also upregulated in response to ETS1 depletion. Additionally, we observed upregulation of the cytokine receptor IFNGR2 (Interferon Gamma Receptor 2) that is critical for innate and adaptive immunity against viral, some bacterial and protozoan infections in response to ETS1 depletion. We also observed increased expression of the interferon-stimulated gene (ISG) TRIM5, a cytoplasmic antiretroviral effector [40] involved in inflammation [41] and autophagy [42]. By contrast, IL27RA, a critical gene for cell-mediated immunity, and TNFRSF25 - a mediator of NF-κB activation and apoptosis, were downregulated.

We also aligned the RNAseq dataset to the HXB2 reference HIV-1 genome to examine the abundance of HIV-1 mapping reads. As expected, HIV-1 mapping reads were rare and coverage across the HIV-1 genome was extremely sparse. For one donor, no HIV-1 reads were observed, but HIV-1 RNA was detected for the other two donors. We observed 1 and 4 unique HIV-1 reads for NT samples and 8 and 7 unique HIV-1 reads for ETS1 depleted cells respectively for Donor 2 and 3 (S2B and S2C Fig), consistent with the hypothesis that ETS1 knockout reactivates HIV-1 expression.

We observed several known and novel candidate regulators of HIV-1 gene expression in the RNAseq dataset. In particular, we observed that expression of the long non-coding RNA MALAT1 (metastasis-associated lung adenocarcinoma transcript 1) was upregulated in ETS1-depleted cells. MALAT1 has been previously reported to promote HIV-1 transcription and infection [43,44], and MALAT1 influences gene expression by several mechanisms, including regulating the recruitment of specific chromatin modifiers such as the Polycomb Repressive Complex 2 (PRC2). Interestingly, one of the top upregulated transcripts in ETS1-depleted cells was another long noncoding gene, AL158151.3, that has recently been reported as being associated with HIV-1 control [45]. Overall, these data demonstrate that ETS1 depletion influences several cellular genes, including some that could have a direct or indirect impact on HIV-1 expression.

### Reactivation of HIV-1 following ETS1 depletion is partially dependent on MALAT1

**Fig 8:**
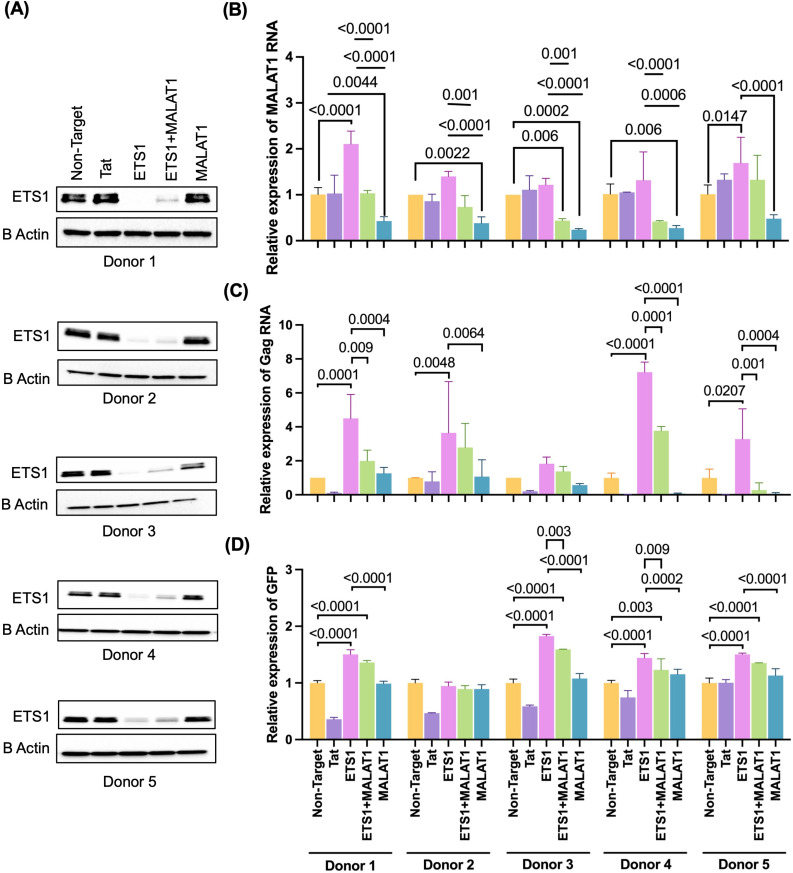
Reactivation of HIV-1 following ETS1 depletion is partially dependent on MALAT1. *In vitro* generated primary latent CD4 T cells were nucleofected with Cas9/gRNA ribonucleoprotein complexes targeting ETS1, MALAT1, or a combination of ETS1 and MALAT1 as described in Fig 6, followed by immunoblotting, flow cytometry and RT-PCR. **(A)** Depletion of ETS1 in nucleofected CD4 T cells was analyzed by western blot. **(B-D)** Relative abundance of MALAT1 RNA expression **(B)**, and Gag RNA **(C)**, and HIV-1 protein expression (**D**) in CD4 T cells nucleofected with Cas9/sgRNAs targeting ETS1, MALAT1, or a combination of ETS1 and MALAT1. MALAT1 and Gag viral RNA expression was normalized to cells nucleofected with NT control. Experiment was conducted in three biological replicates/donors with three technical replicates for each condition. Error bars represent standard deviations, and P values displayed were determined by two-way ANOVA Tukey’s multiple comparisons test.

Having observed a potential impact of ETS1 on MALAT1 expression in HIV-1 infected cells, we next further investigated the impact of ETS1 on MALAT1 and HIV-1 expression. Using our primary CD4 T cell model of HIV-1 latency, we nucleofected cells from five independent donors (2 Males and 3 Females) with RNPs containing gRNAs targeting ETS1, MALAT1, or both ETS1 and MALAT1 in combination. Two weeks post nucleofection of HIV-GFP infected CD4 T cells, the protein level of ETS1 was quantified by immunoblotting (**Fig 8A**). Additionally, we measured the abundance of MALAT1 and HIV-1 Gag RNA in the cells by qRT-PCR (**Fig 8B** and **8C**), and viral protein expression by flow cytometry for eGFP (**Fig 8D**). As expected, ETS1 protein was strongly depleted by ETS1 targeting gRNAs compared to control gRNAs (**Fig 8A**). MALAT1 expression was also significantly depleted in all five donors for cells with gRNAs targeting MALAT1 (**Fig 8B**). Consistent with our RNAseq data, we observed that depletion of ETS1 increased MALAT1 RNA expression within latently infected cells (**Fig 8B**), although the magnitude of the change was variable from donor to donor. ETS1 depletion also caused significant upregulation of HIV-1 RNA in four of the five donors, while a trend towards an increase was observed in the remaining donor (**Fig 8C**). For HIV-1 protein expression, four of the five donors demonstrated a significant increase in GFP after depletion of ETS1, while one donor did not (**Fig 8D**). Depletion of MALAT1 alone did not have a significant effect on HIV-1 expression at the viral RNA or protein level, although viral RNA trended lower in some donors. In contrast, depletion of MALAT1 in latently infected CD4+ T cells in combination with ETS1 knockout decreased HIV-1 reactivation, as determined by quantification of cell associated Gag and eGFP expression (**Fig 8C** and **8D**) although this effect was more pronounced for some donors than others. One donor (donor 2) did not exhibit any reactivation of HIV-1 with ETS1 depletion at the protein (eGFP) level (**Fig 8D**), but exhibited an impact on HIV-1 RNA levels (**Fig 8C**), suggesting that for some donors, additional posttranscriptional blocks continue to inhibit HIV-1 protein expression, even in the presence of ETS1 knockout. These results indicate that ETS1 represses HIV-1 expression at the transcriptional level in latently infected CD4 T cells, and that this effect is partly dependent on ETS1-mediated repression of MALAT1 expression, although donor-to-donor variation exists for this interaction.

### ETS1 depletion enhances transcription-promoting histone modifications at the HIV-1 LTR

**Fig 9:**
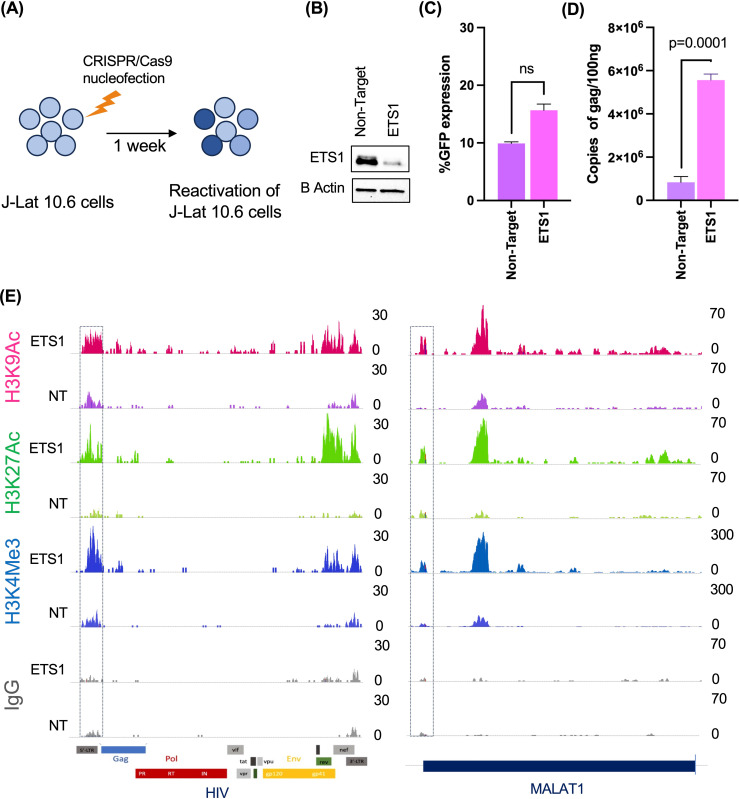
ETS1 depletion increases the abundance of transcription activating histone marks at the HIV-1 LTR. J-Lat 10.6 cells were nucleofected with ETS1 targeting or control non-targeting gRNAs. At 1 week post nucleofection, nucleofected cells were analyzed by Cleavage Under Targets & Release Using Nuclease (CUT&RUN) using antibodies against H3K9ac, H3K27ac and controls H3K4Me3 and IgG. **(A)** Experimental overview. **(B)** Western blot of the ETS1-targeting or control non-targeting (NT) gRNA nucleofected J-Lat10.6 cells for beta actin and ETS1 expression at one- week post nucleofection. **(C)** %GFP+ cells by flow cytometry 1 week post nucleofection with ETS1 and NT gRNA/Cas9 RNPs. **(D)** Abundance of Gag unspliced viral RNA expression in J-Lat 10.6 cells nucleofected with ETS1-targeting or NT control gRNA/Cas9 RNPs. Error bars represent standard deviation, and P values displayed were determined by two-way ANOVA Tukey’s multiple comparisons test. **(E)** Normalized coverage of sequencing reads (aggregated from 3 CUT&RUN technical replicates for each condition) across the HIV-1 reference genome and MALAT1 (chr11:65,498,907-65,506,539) is displayed using Integrative Genomics Viewer version 2.17. The HIV-1 LTR promoter region is denoted in a box. Figure elements prepared using Biorender.

We next sought to further investigate the mechanism by which ETS1 represses expression of the HIV-1 promoter. To examine this, we carried out a Cleavage Under Targets and Release using Nuclease (CUT&RUN) assay [46]. This assay was performed with antibodies against histone marks associated with transcriptionally active promoters, H3K9ac, H3K27ac and H3K4me3, at 1 week post nucleofection of J-Lat 10.6 cells with ETS1-targetting or control non-targeting gRNA/Cas9 complexes (**Fig 9A**). As expected, ETS1 expression was potently depleted from cells that received ETS-1 targeting gRNAs (**Fig 9B**), and HIV-1 expression was reactivated in these cells (**Fig 9C).** A parallel analysis of viral RNA also showed that ETS1 knockout caused significant upregulation of HIV-1 Gag RNA expression within the ETS1 depleted cells (**Fig 9D**) compared to non-targeting (NT) guide RNAs (p = 0.0001; 2-way ANOVA Tukey’s multiple comparisons test). When we aligned the CUT&RUN data to a reference HIV-1 genome, we observed low to moderate abundance of H3K9Ac, H3K27Ac and H3K4me3 at the 5’ LTR in cells nuceleofected with NT gRNAs, reflecting the quiescent nature of the provirus in the latently infected cells. However, in cells depleted with ETS1 H3K9ac, H3K27Ac and H3K4me3 were all more abundantly present across the HIV-1 LTR (**Fig 9E, Left panel**) compared to cells that received non-targeting gRNAs. We also examined the abundance of histone modifications at the MALAT1 promoter (**Fig 9E, Right panel**) after the depletion of ETS1 in latently infected cells. Notably, we found increased abundance of H3K9Ac, H3K27Ac and H3K4me3 marks in the upstream regions of MALAT1 after ETS1 depletion, consistent with the hypothesis that ETS1 represses MALAT1 expression in latently infected cells. Overall, these data suggest that ETS1 represses the addition of transcription activating histone marks at the HIV-1 LTR promoter, thereby likely inhibiting HIV-1 expression.

## Discussion

Complex interplay between numerous viral and cellular transcription factors determines the molecular regulation of HIV-1 transcription, and the HIV-1 promoter contains binding sites for several cellular TFs with either activating and repressive functions [5]. For example, transcription activators that bind to the HIV-1 5′ LTR include Sp1, NF-κB, the AP-1 complex family, NFAT, and the long non-coding RNA MALAT1 [43,47–50]. Transcriptional repressors also bind the HIV-1 5′LTR, including NELF, YY1, ZNF304, and AP4 [51–54]. However, the overall nature of how these factors interact to determine HIV-1 expression is still not well understood and it is likely that additional host factors that regulate HIV-1 exist. Thus, a deeper understanding of the host cell activators and repressors that HIV-1 relies on for both latency or viral gene expression will be required for the effective development and implementation of curative approaches that target HIV-1 expression.

To address this gap, we have employed single-cell technologies (integrated single cell RNA and ATAC sequencing) [6] together with CRISPR-Cas9 lentiviral genomic screening [11] in J-Lat 10.6 to identify a set of novel regulators of HIV-1 expression. In this present study, hundreds of cellular transcripts that were correlated with HIV-1 transcription identified in our previous study [6] were tested for a role in HIV-1 latency by a CRISPR screening strategy. This approach revealed several novel genes involved in latency, including ETS1, TNFAIP3, ZNF740, SAMD12, FOXE3, ZIC5, SMC3 and CDK6. After carrying out further validation in primary CD4 T cells *in vitro* with a small number of selected targets, we confirmed that ETS1 represses HIV-1 expression and maintains latency in primary CD4 T cells.

ETS1 is the prototype of the ETS family of transcription activators and plays an important role in host transcriptional response and in regulation of immune cell function [24], T cell activation and cytokine expression [55], and viral infection [56]. ETS1 has been shown to function either as a transcriptional activator or repressor of numerous genes [57], and is involved in stem cell development, cell senescence and death, and tumorigenesis [58]. Previous work using overexpression to examine the impact of ETS1 on HIV-1 promoter activity in transformed cell lines demonstrated that ETS1 can promote HIV-1 expression, and that an ETS1 binding site exists in the HIV-1 LTR. The divergent behavior of ETS1 with respect to HIV-1 expression at different times post infection could result from different complexes or binding partners of ETS1. Curiously, it has been previously shown that the ETS1 binding site in HIV-1 is not required for activation of the LTR by a truncated version of ETS1, suggesting a possible indirect effect of ETS1 on HIV-1 [31]. In addition to validation studies in an *in vitro* latency model, we also performed ETS1 depletion *ex vivo* in resting CD4 cells isolated from ART-suppressed person with HIV-1 and observed viral reactivation. This discovery clearly demonstrates that ETS1 can play a repressive function in the host cells of the HIV-1 reservoir.

We hypothesize that the depletion of ETS1 affects HIV-1 indirectly by controlling the expression of cellular genes that encode proteins or RNAs that bind to and activate the HIV-1 LTR. Consistent with this hypothesis, RNAseq profiling of ETS1 depleted CD4 T cells identified HIV-1 regulating genes as being significantly enriched within the set of differentially expressed genes. Previous studies have also indicated diverse functions for ETS1 in T cells. ETS1 is essential for the activation of T cells and the production of IFN-γ [59,60]. In a recent study of Helicobacter pylori-associated gastritis, ETS1 expression was shown to be induced by infection and to activate proinflammatory gene expression [61]. ETS1 has also been reported to regulate malignant tumor cell invasion [62] and restrict poxvirus infection [56]. It should be noted that inhibitors for ETS1 are not yet available, commercially or clinically. However, recent studies described novel ETS1 inhibitors and their role in antitumor activity against hepatitis B/C virus (HBV/HCV) induced hepatocellular carcinoma [62], lymphoma [63] and vessel regression [64].

Interestingly, in our RNA-Seq data, we observed that ETS1 depletion in resting CD4 T cells upregulates MALAT1, a known positive regulator of HIV-1 expression. This observation suggests a potential regulatory relationship between ETS1 and MALAT1 in the context of HIV-1 infection. The association of MALAT1 expression with HIV-1 transcription has been reported in several studies [43,65,66]. Notably, MALAT1 interacts with the PRC2 complex and may protect the HIV-1 LTR from PRC2-mediated epigenetic silencing [65,67]. Additionally, depletion of ETS1 caused the upregulation of cellular genes that are involved in ATP-dependent chromatin remodeling. Chromatin remodelers play a critical role in regulating multiple cellular processes, such as DNA repair and gene expression, creating nucleosome-depleted regions by promoting the exchange of histones [68]. Such chromatin remodeling complexes are also involved in regulating the chromatin architecture at the HIV-1 LTR thereby regulating LTR transcription [69]. Alterations to chromatin remodeling may thus contribute to the latency regulating activity of ETS1. Combined depletion of MALAT1 with ETS1 indicated that this attenuated the upregulation of viral gene expression induced by ETS1 knockout, suggesting that the impact of ETS1 knockout on HIV-1 is partly dependent on MALAT1 upregulation, although the magnitude of this effect was variable. Indeed, the binding of MALAT1 and ETS1 was reported in a recent tumor study [70]. The interplay between MALAT1 and ETS1 has not been previously reported for HIV-1 infection, and we propose that this represents a novel indirect mechanism of HIV-1 regulation by ETS1. Notably, ETS1 is polymorphic in humans with several different alleles being present at high frequency [71]. These genetic difference may contribute to the variable impact of ETS1 on HIV-1 and MALAT1 in primary CD4 T cells.

We observed that depletion of ETS1 in latently infected cells led to increased H3K9ac, H3K27ac and H3K4me3 at the viral LTR. H3K9ac, H3K27ac and H3K4me3 are all histone modifications that mark regions of active gene transcription. In particular, the presence of acetylation marks on chromatin is typically associated with open, accessible regions that promote gene expression [27,29,72], and when these modifications are absent or reduced, the HIV-1 provirus is typically silenced and considered latent [73]. Thus, the increase in acetylation at the LTR in latently infected cells depleted with ETS1 suggests that ETS1 inhibits the activity of histone acetyltransferases at HIV-1 LTR promoter, thereby blocking the addition of these acetylation marks and limiting expression of the HIV-1 latent reservoir.

Overall, these data indicate that ETS1 is an important regulator of HIV-1 latency and could represent a useful target for latency reversing strategies. Additional work to further reveal the precise mechanisms by which ETS1 mediates positive and negative regulation of HIV-1 will also help to direct the development of novel LRAs that target ETS1.

## Methods

### Ethics statement

Cells from people living with HIV-1 were derived from the UNC clinical cohort and participants provided written consent for analyses of their HIV-1 reservoir (Study #: 08-1575). The study was reviewed and approved by the UNC Chapel Hill Institutional Review Board.

### Cell lines

The latently infected Jurkat T-lymphocyte cell lines J-Lat 10.6 [14,15], 2D10, and Jurkat-N6 were maintained in Gibco RMPI medium (Thermo Fisher Scientific), supplemented with 10% fetal bovine serum, 2 mg/mL L-glutamine, sodium pyruvate, penicillin-streptomycin and 10mM HEPES. Cells were cultured at 37 °C with 5% CO_2_. The J-Lat 10.6 [14,15] cells are a derivative of Jurkat cells that was infected with a pseudotyped HIV-1 strain, HIV/R7/E−/GFP. The human embryonic kidney HEK293T (ATCC; CRL11268) cell line that was used for transfection was maintained in DMEM complete medium (Gibco), supplemented with 10% fetal bovine serum, and penicillin-streptomycin.

### Clinical samples and primary CD4 T cell model of HIV-1 latency

Ficoll-Paque (Thermo Fisher Scientific) was used to purify peripheral blood mononuclear cells (PBMCs) from leukapheresis donations obtained from durably ART-suppressed people with HIV-1 (PWH). Total CD4 T cells were isolated from PBMCs using an EasySep Human CD4+ T Cell Isolation Kit (#17952, StemCell Technologies) following the manufacturer’s protocol. CD4 T cells isolated from ART-suppressed people with PWH were cultured in RPMI media supplemented with 10 units per mL of IL-2. To generate primary CD4 T cells that were latently infected with HIV-1 *ex vivo*, we used a latency model that we have previously reported [6,27,30]. Briefly, CD4 T cells from seronegative donors were activated with anti-CD3/CD28 beads (Life Technologies) at 1M cells per mL for 48h, then infected with a replication defective HIV-1 reporter clone, NL4-3-△6-drEGFP-IRES-Thy1.2 that had been pseudotyped with the Vesicular Stomatitis Virus G protein (VSV-G) [6,26]. At 48h post infection, infected (GFP+) cells were enriched by magnetic separation of Thy1.2+ cells, then cultured for up to two additional weeks in RPMI media (Thermo Fisher Scientific), supplemented with 10% fetal bovine serum, 2 mg/mL L-glutamine, sodium pyruvate, penicillin-streptomycin and 10mM HEPES. Primary CD4+ T cells were additionally supplemented with 20U/mL IL-2 and 4ng/mL IL-7. Cells were cultured at 37 °C with 5% CO_2_.

### gRNA library generation

Guide RNAs for the 354 HIV-1 correlated genes were generating using GUIDES [74], CHOPCHOP [75] and Synthego gRNA generation algorithms. For each gene, 8 individual gRNAs were generated. The library was supplemented with 150 non-targeting controls (NTCs), to make a total gene library size of 2946 guides (Twist Biosciences, San Francisco, CA). The full list of genes and corresponding guides is available in S4 Table. DNA sequences corresponding to gRNAs were cloned into the HIV-CRISPR vector as previously described [12]. Briefly, BsmBI overhangs were PCR amplified on the guide RNA library and cloned into a BsmBI-digested (New England Biolabs, R0134S) HIV-CRISPR vector by Gibson Assembly (New England Biolabs, E2611S). Representation of guide RNAs in HIV-CRISPR vector was validated by next generation sequencing.

### HIV-CRISPR library transduction and screen

The pool of HIV-CRISPR vectors containing the gRNA library was transfected into HEK293T cells. Transfection was performed with the library containing HIV-CRISPR vector (667 ng) along with psPax2 (Gagpol, Addgene, 12260, 500 ng) and MD2.G (VSVG, Addgene, 12259), in serum-free DMEM and the TransIT-LT1 reagent (Mirus Bio, MIR2305). Virus was harvested and J-Lat 10.6 cells were transduced with the TxLatent library at a multiplicity of infection of 0.2. After 13 days of puromycin selection, cells were treated with DMSO (ThermoFisher Scientific, D139-1), vorinostat (500nM, SelleckChem, S1047), AZD5582 (10nM, MedChemExpress, HY-12600), prostratin (75nM SigmaAldrich, P0077), or iBET151 (75nM, SelleckChem, S2780).

### CRISPR/Cas9-mediated knockout of host genes in primary CD4 T cells and latently infected cell lines

Pre-designed CRISPR RNAs (crRNAs) against ETS1 (CAAGACGGAAAAAGTCGATC, CAGAAACCCATGTTCGGGAC, and CGAGAAAGCAGTCTTTACCC), and other human gene targets, CDK6, TNFAIP3, ZNF740, SAMD12, FOXE3, ZIC5, SMC3 (S5 Table) and/or HIV-1 Tat and scrambled guides (non-target control, NT) were purchased from Integrated DNA Technologies (IDT). Custom crRNAs for MALAT1 (ACTTCTCAACCGTCCCTGCA, CTGGTTCTAACCGGCTCTAG, and CCTGACGCAGCCCCACCGGTT), reported previously [76,43], were purchased from IDT. crRNAs with superior off-target scores were selected and used for the experiment. Targeting sequences of crRNAs are provided in S5 Table. Annealing of crRNA/tracrRNA, and preparation of CRISPR/Cas9 ribonucleoprotein complexes (RNPs), and electroporation was performed as described previously [6]. Briefly, two or three crRNAs targeting different regions of the target were multiplexed for more efficient target knockout. For each electroporation, 2-3x10^6^ HIV-GFP infected primary CD4 cells and latently infected cell lines (2D10, J-Lat 10.6, and Jurkat-N6 cells) were washed twice with phosphate buffered saline (PBS) at a speed of 90 g for 10 min then resuspended in nucleofection buffer P3 (Lonza). The resuspended cells were combined with crRNP complexes and were immediately transferred into the cuvette of the P3 Primary Cell Nucleofector Kit (Lonza; V4SP-3096) and electroporated using code CM-137 on the Lonza 4D-Nucleofector. P2 buffer and program EH100 was used for latently infected primary CD4 cells, and for cells isolated from PWH program. Electroporated cells were resuspended with 200μL of prewarmed supplemented RPMI media with IL-2 (20U/mL) and IL-7 (4ng/mL) and expanded in a 37°C incubator for subsequent experiments. The cells were maintained at ~1x10^6^ cells/mL with fresh media supplemented with IL-2 and IL-7 every 2-3 days.

### Quantification of gene expression using quantitative-PCR

RT-qPCR for HIV-1 Gag RNA and MALAT1 RNA was performed as previously described [30]. Briefly, RNA was extracted from cells harvested at three days or two weeks post nucleofection using the RNEasy plus kit (Qiagen) as per the manufacturer’s instructions and quantified by nanodrop. 1μg of RNA for cells from PWH and 100ng of RNA from *in vitro* primary CD4 T cells were reverse transcribed and amplified using Fastvirus (Thermo, Waltham, MA) and primer sets for HIV-1 Gag RNA (GAG-F: ATCAAGCAGCCATGCAAATGTT, GAG-R: CTGAAGGGTACTAGTAGTTCCTGCTATGTC, GAG Probe: FAM/ZEN-ACCATCAAT GAGGAAGCTGCAGAATGGGA-IBFQ). A Gene expression assay primer/probe kit from Thermo-Fischer was used for the quantification of MALAT1 (Hs00273907_s1, Thermo Fischer Scientific). Reactions were performed in 96-well plates using the Quant Studio 3 Real-Time PCR system (Applied Biosystems, Foster City, CA) real time thermocycler with a cycling parameters of 5 min reverse transcription step at 50°C, 95ºC for 20 sec for Taq activation, followed by 40 cycles of 95ºC (3 sec.), and 60ºC (30 sec.). All qPCR reactions were performed in triplicate. Normalized relative expression levels were calculated using the Prism software version 10.1.1 (GraphPad).

### RNAseq analysis

Total RNA was harvested from nucleofected CD4 cells from PWH using a RNEasy plus kit (Qiagen). The quality of the RNA was assessed using an Agilent 2100 bioanalyzer with the RNA 6000 Pico Chip (Agilent Technologies, Amstelveen, The Netherlands). The quantity of RNA was calculated using Qubit (Thermo Fisher) and the integrity of the RNA was assessed as RNA integrity number (RIN) scores. RNAseq libraries were prepared using the KAPA Total RNA library prep kit, followed by 50 bp paired-end sequencing on a Nextseq2000 P2 flow cell. 70-90 million reads were obtained per sample. To analyze the data, the raw reads were first filtered by FastQC and CutAdapt to remove low quality reads. STAR version 2.7.1a was then used to align the trimmed reads to integrated human genome (GRCh38) and HIV-1 genome (HXB2) followed by featurecounts to quantify transcripts [77]. Differentially expressed genes were then identified by DESeq2 [78]. Database for Annotation Visualization and Integrated Discovery (DAVID) was used for functional annotation bioinformatics microarray analysis to determine the functional enrichment [79]. Bam files generated from RNASTAR were used as input for integrative genome viewer (IGV) to visualize gene transcript abundance [80].

### Immunoblotting

To extract cellular protein for western blotting, cells were lysed using RIPA buffer. Lysates were resolved on a NovexTM WedgeWellTM 4–20% Tris-Glycine gel (Invitrogen; XP04202BOX) and transferred to nitrocellulose membranes (Invitrogen; IB23002). Blocking was performed for 1h at room temperature using 5% milk in Tris-buffered saline (TBS). Immunoblotting was performed using the primary antibodies, ETS1 (Cell signaling Technology; 14069), CDK6 (abcam; ab310613), ZIC5 (Sigma Aldrich; SAB2107906), SMC3 (abcam; ab9263), ZNF740 (Thermo Fisher; 25411-1-AP), TNFAIP3 (abcam; ab92324), SAMD12 (abcam; ab121831), FOXE3 (Thermo Fisher; 55301-1-AP) at 1:1000 dilution, and Beta actin (abcam; ab49900) at 1:10000 dilution. Membranes were washed with 0.1% TBS-tween (TBST) 3 times for 10 min each. The following secondary antibodies were used at 1:10000 dilution: donkey anti-rabbit IgG-HRP (Novex; A16035), and donkey anti-mouse IgG-HRP (Novex; A16011). Membranes were developed with ECL Prime Western Blotting detection reagents (Cytiva; RPN2232) and visualized on a BioRad Chemidoc MP Imaging System.

### CUT&RUN

One week post nucleofection of J-Lat 10.6 cells with ETS1 or NT control targeting CRISPR RNPs, cells were harvested in triplicate and CUT&RUN was carried out following as previously described [27,29] using a commercially available kit (Epicypher; 14-1048). H3K9Ac (CST; 9649S) and H3K27Ac (Epicypher; 13-0059) antibodies were used to map acetylated chromatin, while H3K4Me3 and IgG antibodies, included in the Epicypher kit, served as controls. An NEBNext ultra II DNA library prep kit (New England Biolabs; E7645) was then used for the construction of Illumina sequencing libraries. Libraries were sequenced on the Illumina NextSeq2000 P3, obtaining approximately 200 million paired-end reads (50 × 50 nucleotides) on average. Adaptor trimmed paired-end FASTQ files were aligned to the combined human (Hg38), E. coli (MG1655 reference genome) and J-Lat 10.6 HIV-1 reference genome (GenBank: MN989412.1) using Bowtie2. BamCoverage was used to generate bigwig files from E. coli spike-in DNA normalized bam files, followed by visualization using the Integrative Genomics Viewer (IGV).

### Data and statistical analysis

Flow cytometry data were analyzed using FlowJo version X10.0.7r2 (FlowJo LLC; Ashland, OR, USA). Uninfected primary CD4 cells were used as a control for GFP gating for CRISPR knockout experiments. GraphPad Prism v. 10.1.1 (GraphPad; San Diego, CA, USA) was used for plotting the data. For J-Lat 10.6 cells, background GFP from unstimulated cells was subtracted from all samples prior to normalizing. All data are presented with error bars as mean ± standard deviations from at least 3 independent experiments. Statistically significant differences in provirus expression were determined by two-way ANOVA Tukey’s multiple comparisons test, with a p value <0.05 considered significant.

## Supporting information

S1 Fig Reverse transcriptase assay. Reverse transcriptase assay was carried on supernatant out to detect release of HIV-1 particles from J-Lat 10.6 cells after stimulation with latency reversing agents.
(TIFF)

S2 Fig IGV display of ETS1 read disruption and HIV-1 reads in ETS1 nucleofected CD4 T cells from PWH. (**A**) ETS1 mapping reads for each of the two conditions (ETS1 targeted and NT control) are visualized using the Integrative Genomics Viewer (IGV). The change in read depth between gRNA targeting sites is a characteristic of indels that cause larger disruption of ETS1 transcript due to CRISPR editing. The read disruption is present in all the three of the ETS1 nucleofected cells, approximately half the reads and none of the reads in the NT control. Position, strand and sequence of the gRNAs for the ETS1 target is provided at the bottom. (**B**) Visualization of HIV-1 mapping reads from bulk RNAseq data for ETS1-targeting and NT control across the HIV-1 genome. HIV-1 reads were undetected in sample (Donor 1). **(C)** Number of unique HIV-1 mapping reads identified in each sample after alignment to an HIV-1 (HXB2 strain) reference genome.
(TIFF)

S1 Table List of genes targeted by TxLatent gRNA library. (XLSX)

S2 Table Enrichment scores for genes targeted by TxLatent in J-Lat 10.6 cells. (XLSX)

S3 Table Differentially expressed genes following ETS1 depletion in CD4 T cells from three PWH on ART. (XLS)

S4 Table List of genes and corresponding guides that comprise the TxLatent Library. (DOCX)

S5 Table List of gRNAs targeting different transcripts/transcription factors in primary CD4 T cell latency model. (DOCX)
