# Supplementary figures and images for: A targeted CRISPR screen identifies ETS1 as a regulator of HIV-1 latency
Source: PLoS Pathog. 2025 Apr 8;21(4):e1012467. doi: 10.1371/journal.ppat.1012467 (PMC12005537; doi:10.1371/journal.ppat.1012467)

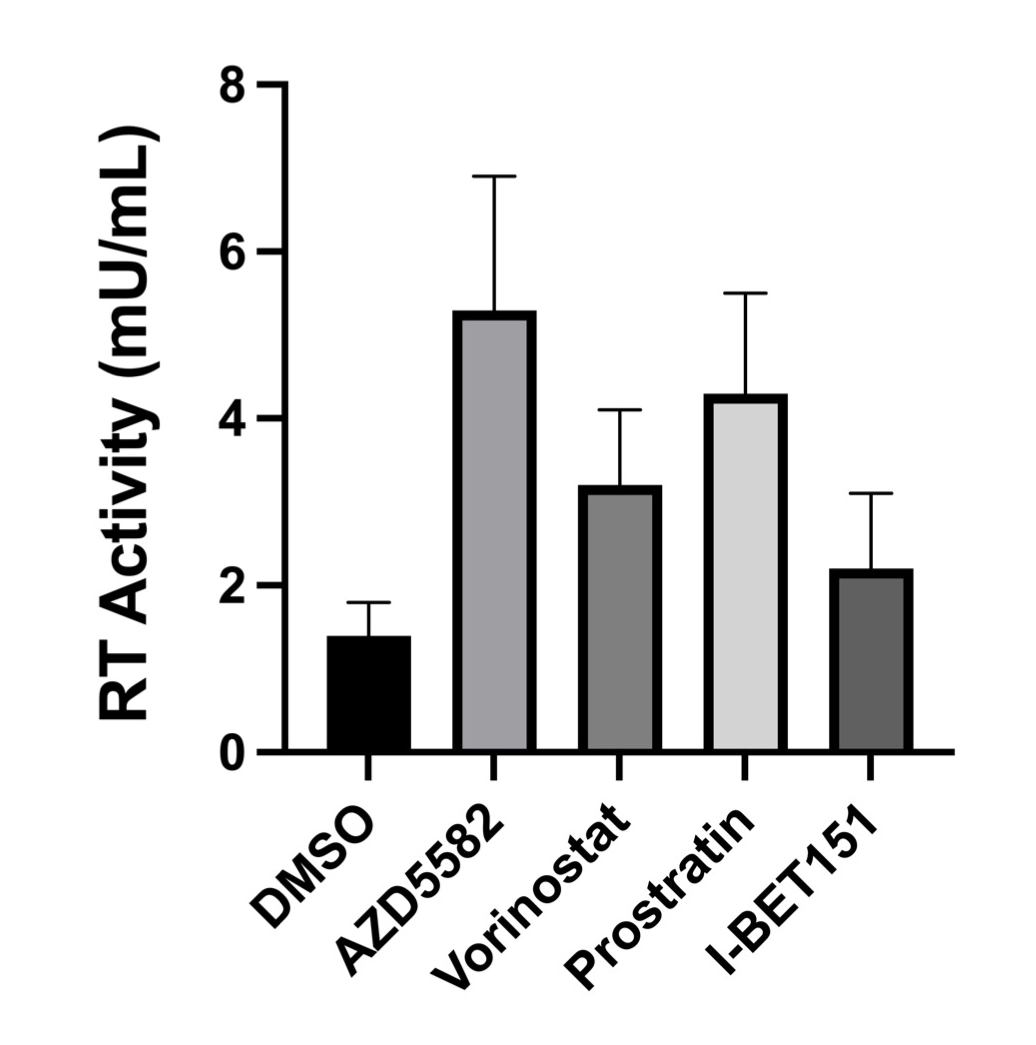

Supplement: S1 Fig — Reverse transcriptase assay was carried on supernatant out to detect release of HIV-1 particles from J-Lat 10.6 cells after stimulation with latency reversing agents. (TIFF) [file ppat.1012467.s001.tiff]

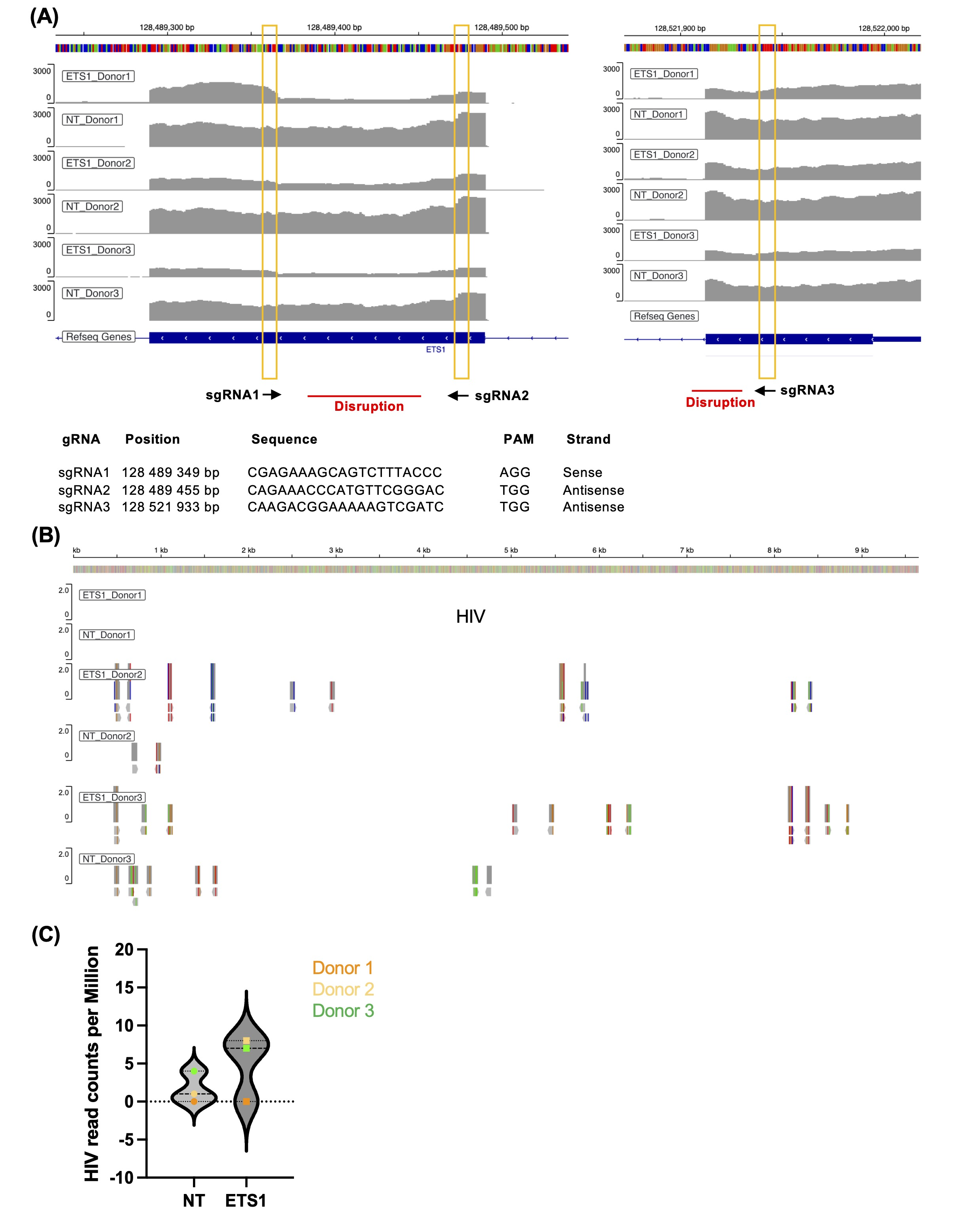

Supplement: S2 Fig — (A) ETS1 mapping reads for each of the two conditions (ETS1 targeted and NT control) are visualized using the Integrative Genomics Viewer (IGV). The change in read depth between gRNA targeting sites is a characteristic of indels that cause larger disruption of ETS1 transcript due to CRISPR editing. The read disruption is present in all the three of the ETS1 nucleofected cells, approximately half the reads and none of the reads in the NT control. Position, strand and sequence of the gRNAs for the ETS1 target is provided at the bottom. (B) Visualization of HIV-1 mapping reads from bulk RNAseq data for ETS1-targeting and NT control across the HIV-1 genome. HIV-1 reads were undetected in sample (Donor 1). (C) Number of unique HIV-1 mapping reads identified in each sample after alignment to an HIV-1 (HXB2 strain) reference genome. (TIFF) [file ppat.1012467.s002.tiff]
