# Supplementary material for: A targeted CRISPR screen identifies ETS1 as a regulator of HIV-1 latency
Source: PLoS Pathog. 2025 Apr 8;21(4):e1012467. doi: 10.1371/journal.ppat.1012467 (PMC12005537; doi:10.1371/journal.ppat.1012467)
Supplement: S5 Table — (DOCX) [file ppat.1012467.s007.docx]

**Supplementary table 5.** List of gRNAs targeting different transcripts/transcription factors in primary latent cell model.

| **Target** | **Sequence** | **Position** | **Strand** |
| --- | --- | --- | --- |
| CDK6 | GACCACGTTGGGGTGCTCGA | 92833092 | Sense |
|  | AACACTCCAGAGATCCACGG | 92671457 | Sense |
|  | CTGGACTGGAGCAAGACTTC | 92671488 | Sense |
| ETS1 | CAAGACGGAAAAAGTCGATC | 128521933 | Antisense |
|  | CAGAAACCCATGTTCGGGAC | 128489455 | Antisense |
|  | CGAGAAAGCAGTCTTTACCC | 128489349 | Sense |
| TNFAIP3 | TGAAGTCCACTTCGGGCCAT | 137875741 | Antisense |
|  | AACCATGCACCGATACACAC | 137871349 | Sense |
|  | TCAACTGGTGTCGAGAAGTC | 137871474 | Sense |
| ZNF740 | TAGGTGGTAACTGCTCCGAA | 53186032 | Antisense |
|  | GAACCAAACTCACACCTGCT | 53184916 | Antisense |
|  | GAGCCAGATTGCCAGCAAGC | 53184964 | Sense |
| SAMD12 | GGTGAAGCTATCTAAACCGG | 118439933 | Antisense |
|  | AGGAACTCCCAAGCGACTGC | 118580737 | Antisense |
|  | GTTGTAAGATGTGCTGCCGG | 118379616 | Sense |
| FOXE3 | GCGGTAGAAGGCAAAGCGTT | 47416622 | Antisense |
|  | CGTTGAGCGTGAGATTGTGG | 47416674 | Antisense |
|  | CAAAGCGTTCGGTGATGAAG | 47416611 | Antisense |
| ZIC5 | CATGCGGCAGCCAATCAAGC | 99970495 | Antisense |
|  | CGCGACCAAGGCTGCAGCAC | 99971288 | Sense |
|  | GGTGCCGAAAGTTTTGGAGC | 99970364 | Sense |
| SMC3 | TAGAGTGTATGACGAACGAA | 110580968 | Sense |
|  | TCTGCTAAGCGAGAGACTAG | 110582564 | Sense |
|  | GGATAAAATGAGACGAGCCC | 110582022 | Sense |
